# Which needs do children, adolescents, and young adults with ME/CFS and their parents express and feel? - Qualitative study in Germany

**DOI:** 10.1186/s12889-026-29549-6

**Published:** 2026-09-29

**Authors:** Alissa Kircher, Sandra Mayer-Huber, Maria Eberhartinger, Lorenz Mihatsch, Christian Apfelbacher, Silvia Stojanov, Uta Behrends

**Affiliations:** 1 Munich Chronic Fatigue Center for Young People (MCFC), Paediatrics, TUM School of Medicine and Health, Technical University of Munich, Munich, Germany; 2 Institute of Social Medicine and Health Systems Research, Medical Faculty, Otto von Guericke University Magdeburg, Magdeburg, Germany; 3 Munich Chronic Fatigue Center for Young People (MCFC), Child and Adolescent Psychosomatics, TUM School of Medicine and Health, Technical University of Munich, Munich, Germany; 4 Division of Pediatric Psychosomatic Medicine, Department of Pediatrics and Adolescent Medicine, KJF Klinikum Josefinum, Augsburg, Germany

**Keywords:** Myalgic encephalomyelitis, Chronic fatigue syndrome, ME/CFS, Paediatric, Children, Adolescents, Parents, Needs analysis, Treatment concept, Focus groups, Adaptive diary approach

## Abstract

**Background:**

Myalgic encephalomyelitis/chronic fatigue syndrome (ME/CFS) is a complex, multi-system disease that significantly deteriorates the quality of life for those affected. There is no established effective treatment, and a lack of research on what is needed by severely affected young people with ME/CFS and their caring parents to cope with the disease. The aim of this study was to investigate the needs of young people affected and their parents in order to subsequently build a tailored treatment model for this age group upon this basis.

**Methods:**

Needs were explored using topic-guided focus groups and an adapted diary method. Patients participated either in an online focus group with five sessions discussing needs related to five topics (sports/physical activity/mobility, self-care, mental activity, social contacts, and school/vocational training/study/profession) or in the adaptive diary approach, receiving and answering the same stimuli and questions in the format of voice and text messages. Additionally, focus groups with parents comprised two sessions each, targeting needs of their children and themselves, respectively. Data were analysed by reflexive thematic analysis.

**Results:**

We included 14 young people with ME/CFS (aged 12 to 25 years), of whom five participated in a focus group and nine in the adaptive diary approach. Eighteen parents took part in three focus groups, including three couples. In the patient sample, four themes of unmet needs were identified: (i) not being left alone with the disease, (ii) flexibility and understanding of healthcare providers and authorities, (iii) having to explain oneself less to others, and (iv) appreciation of the family filling part of the healthcare gap. Among parents, the following four themes were revealed: (i) not being left alone with the disease, (ii) health care adapted to ME/CFS, (iii) knowledge, understanding, and acceptance of ME/CFS, and (iv) confidence in dealing with ME/CFS.

**Conclusions:**

The needs of young people with ME/CFS and their parents, respectively, overlap clearly and demonstrate the importance of a needs-based adaptation of healthcare services.

**Supplementary Information:**

The online version contains supplementary material available at https://doi.org/10.1186/s12889-026-29549-6.

## Introduction

Myalgic encephalomyelitis/chronic fatigue syndrome (ME/CFS) (ICD-10 GM G93.3, ICD-10 CM G93.32) is a chronic, debilitating multi-systemic disease that can occur at any age, with two age peaks of onset at 11–19 and 30–39 years [1]. Experts estimated that approximately 140.000-310.000 people in Germany were affected by ME/CFS before the corona virus disease 2019 (COVID-19) pandemic, including 70.000–90.000 school-aged children and adolescents [2]. However, a considerable number of cases without a diagnosis are assumed [3]. Affected persons suffer from mental and/or physical pathological exhaustion (fatigue), deterioration of symptoms after everyday activities (post-exertional malaise, PEM), sleep problems, pain, as well as neurocognitive, autonomic, neuroendocrine and/or immunological manifestations [4]. Up to 25% of patients are severely affected and bound to home or bed [5, 6].

ME/CFS in young people mostly follows an infection, e.g. infectious mononucleosis or COVID-19 [7, 8]. The diagnosis is based on clinical criteria and thorough differential diagnostics [9, 10]. The duration of the disease is variable. The definition of recovery varies across studies, making direct comparisons challenging. In addition, children generally have a substantially better prognosis than adult patients. Long-term follow-up data of a large paediatric sample showed roughly one-third recovering after five years and roughly two-thirds after ten years [11]. In a study of adults with the condition, the reported rates of recovery and clinical improvement were 8.3% and 4.8%, respectively [12]. Health-related quality of life (HRQoL) with ME/CFS is lower than with other severe chronic diseases [13]. ME/CFS patients experience great distress, secondary psychological reactions, as well as stigmatization [14, 15]. Consequently, the personal, family, social and economic damages are enormous [16, 17]. Mortality is increased compared to the general population [18].

Although causal treatment approaches do not yet exist, recovery can be supported and secondary damage prevented with dedicated symptom-oriented care and rest [15]. As the treatment of this patient group is complex and costly, there are only few healthcare services for ME/CFS patients in Germany, especially access to care for homebound or bedbound patients is missing [19]. Moreover, severely or very severely affected ME/CFS patients are rarely included in research projects, which results in a specific lack of knowledge about this group of patients [6 , 20].

To implement adequate outreach, healthcare structures data on medical and psychosocial needs of homebound patients and their caregivers is required. Studies on adult patients have been performed to identify the needs of ME/CFS patients and to develop new healthcare structures [21]. A review of 32 studies by Drachler et al. from 2009 identified three main needs in the context of ME/CFS: (i) understanding symptoms and receiving a diagnosis, (ii) respect and empathy from service providers, and (iii) positive attitude and support from family and friends [22].

These studies underscore the significance of a comprehensive approach to both physical and psychosocial factors in illness development and management: A community-based management program study for adolescents and their families demonstrated significant improvements in physical and psychological functioning [23]. Social isolation, loss of educational opportunities, and challenges in obtaining adequate public support were highlighted as significant hurdles [24]. Further studies stressed the importance of diagnosis acceptance, support from families and physicians, and structured interventions for symptom management and social integration [25, 26]. Findings from a recent qualitative workshop study with adult patients emphasized the importance of early support interventions, training for healthcare professionals, and broader public education [27]. None of these studies were conducted in Germany, and only half of them were conducted in Europe, predominantly in the United Kingdom (UK). Only six of these studies included children and/or adolescents, most of them published many years ago and before the COVID-19 pandemic. Apart from the studies included in the review by Drachler et al., two publications in 2015 and 2018 by Parslow et al. emphasized the complexity of the needs of affected children and adolescents, the necessity of tailored support systems, including the family, and the importance of contextual factors as symptoms fluctuate depending on the activity and influence of the surroundings [28, 29]. Their systematic review of qualitative studies highlighted the importance of acknowledging the ME/CFS diagnosis, delivering an activity management strategy, and of improving communication between health care providers [30]. A more recent study by Sommerfelt et al. [31] highlighted the problems and needs particularly relevant to ME/CFS patients in Norway. In particular, respondents expressed the need for a broader acceptance and a better understanding of the disease by society and the healthcare system, as well as better practical assistance in everyday life by healthcare professionals [31].

The mentioned studies hardly took the family aspect into account [17, 22]. Since parents usually take care of their sick children, it is essential to investigate parental needs when setting up new care structures. None of the existing studies included the parents’ focus on needs, but some aspects were investigated from which potential needs could be derived. Some studies analysed the psychological stress of parents caused by their children’s illnesses [17, 32],which could lead to the assumption that parents need psychological support. Other studies emphasize the financial burden of the disease on families [17, 33],which suggests a need for financial support. Difficulty in accessing care is the focus of another study [34], suggesting a need for easier access to care. Baken et al. explored the experiences of parents in New Zealand dealing with ME/CFS and found the already mentioned psychological stress of parents, as well as the little knowledge and understanding, being the most difficult topics [35]. It remains to be investigated whether this experience of the parents from New Zealand correlates with the needs of parents in Germany.

To conclude, none of the mentioned studies was conducted in Germany, and most of the studies were conducted before the COVID-19 pandemic. Some highlight singular aspects but lack a comprehensive and deep understanding of the needs of young patients with ME/CFS. The needs of caring parents, which can provide a valuable addition to the understanding of the necessary support needed by the patient group, have rarely been investigated in prior studies.

Against this background, this study aimed to explore the needs of adolescent and young adult patients, including the perspective of their parents.

## Methods

This study is reported according to COREQ.

### Recruitment and participants

This study was conducted as part of the study “ME/CFS Kids Bavaria” between 01.07.2022 and 31.12.2023 at a tertiary paediatric university hospital in Germany. Children, adolescents, and young adults were recruited during or after personnel visits to our ME/CFS outpatient clinic. Furthermore, parents of affected patients were enrolled in the study to assess their perceptions of their children’s needs and to explore their own needs arising from their caregiving role within the family. The participants were recruited face-to-face, by phone or via email. Written informed consent was obtained from all patients, from legal guardians if patients were aged less than 18 years, and from participating parents.

We used convenience sampling with defined criteria: Study inclusion criteria for participating patients were age 12–24 years, residence in Bavaria, and ME/CFS diagnosis at the Munich Chronic Fatigue Center for Young People (MCFC). The same criteria had to be fulfilled by patients represented here by their parents. For being diagnosed with ME/CFS, patients had to meet at least one PEM-based clinical case definition, the Canadian consensus criteria [7], the Institute of Medicine (IOM) criteria [4], and/or, in the case of adolescents and children, one of two paediatric case definitions [15, 36]. Some patients were included after partial recovery from ME/CFS. Exclusion criteria for patients were any documented acute mental illness, insufficient knowledge of the German language, and/or a too high psychological or physical extra burden by the study as judged by the study team together with the family.

### Procedure/ data collection

Patients and parents were distributed into focus groups. Severely affected patients were offered a participation via adaptive diary method. All participants received Patient-Reported Outcome Measures (PROMs). Sociodemograpic data were additionally collected from participating patients and parents. Given the small sample size of our samples and the highly preselected cohort, only parts of the data were included in the manuscript. Additional data of parents were included in Supplementary material- Sociodemographic data of the parental sample. The data of patients about educational background were excluded from the publication in order to protect participant confidentiality.

### Descriptive analysis of the study population via patient-reported outcome measures

In order to describe the study population, all participants (patients and parents) received a set of questionnaires at the end of their participation in focus groups or adaptive diary method. This time point was chosen to reduce extra challenges preceding the focus groups or the diary-based evaluations. Patients were allowed to answer the questionnaires at any time individually needed. Parents completed the PROMs with reference to their own physical and mental health status and not as a proxy assessment of their affected children. The PROMs were included to provide descriptive information on sample characteristics.

#### Bell score

The Bell CFIDS disability scale (Bell Score) is a widely used instrument in the diagnostics of ME/CFS [37, 38]. It is a questionnaire to assess daily functioning on a scale from 0 to 100, with 100% indicating normal daily functioning and 0% indicating confinement to bed. Only patients received this questionnaire.

#### Short form 12 health survey questionnaire

The Short Form 12 Health Survey Questionnaire (SF-12) is a consistent and reliable measure of the HRQoL [39]. It includes eight subscales: Physical Functioning, Role-Physical, Bodily Pain, General Health, Vitality, Social Functioning, Role-Emotional, and Mental Health. For example, participants were asked to rate their general health and to indicate the extent to which physical pain or emotional problems interfered with their usual daily activities. The physical component scale (PCS) and mental health component scale (MCS) were calculated using the standard scoring algorithm from *Hogrefe*, based on the German norm population from 1998 [40]. The scores of the subscales can range between 0 and 100, with higher scores indicating a better health status.

#### Hospital anxiety and depression scale german version

The Hospital Anxiety and Depression Scale German version (HADS-D) is a well-validated questionnaire to screen for anxiety and depression in patients with physical illnesses or physical complaints, comprising 14 items, each rated on a 4-point Likert scale [41]. Sum values can indicate possible emotional disorders or reflect probable cases of morbidity. For adolescents, adapted values are used in order to minimize false negatives or false positives [42].

### Focus groups

The focus group design was chosen as a well-established method in health research that allows to explore needs [43]. Focus groups are moderated group discussions and can be varied in duration, which is an essential requirement in the context of ME/CFS. Regarding the impaired mobility of most patients, an online format was chosen. All focus groups were moderated by a physician (AK) and co-moderated by a psychologist (SMH). Apart from the researchers and the participants, no other person participated in the video sessions.

Patients took part in one focus group comprised of five sessions, dealing with five different topics: sport/physical activity/mobility, self-care, mental activity, social contacts, and school/vocational training/study/profession and was moderated by a physician and co-moderated by a psychologist. The duration of the sessions for patients was limited to a maximum of one hour due to patient stress. Parents were best off assigned to three groups (AK, ME) based on their children’s age, and each group attended two 1,5-hour sessions between September and February 2023. One session focused on the needs of the affected children according to the parents’ perspective, and the other session covered the needs of the parents themselves, focusing on the topics of communication and the impact of the disease on families. Two groups started with a focus on the children’s needs, and in one group, the order of the topics was reversed to avoid a sequential bias. In all groups, the topics were introduced via stimuli seen on the screen to stimulate a discussion on the respective topic. The audio was recorded for later analysis, and simultaneously with the discussion, the answers of participants were thematically noted on the screen visible to participants.

### Adaptive diary method

Severely affected patients who wanted to participate but were not able to tolerate the cognitive and/or physical exertion associated with the setting were offered an asynchronous survey between January and April 2023 using an adapted diary. Participants received the same stimuli as the focus group members via an audio or text file at the beginning of each week and were given one week to answer the respective topic by voice or text message according to their own energy level. To avoid a sequence bias, the order of topics was changed for each participant, with the exception of self-care never being the first topic, as this is considered a very intimate subject. Participants sent their messages to a data room, fulfilling state-of-the-art data protection requirements.

### Data analysis

Maslow described the importance of exploring deeper motivations behind the stated needs, as different needs can belong to the same subconscious category of needs, and these underlying needs are of higher stability [44]. Wright et al. differentiate between normative needs (capacity to benefit), expressed needs (what patients ask for) and felt needs (what patients want) [45]. Our analysis combines both concepts and concentrates on the deeper level of needs and aims not only to identify the expressed needs but also the felt needs behind them. This approach aligns with Drachler’s quality assessment criteria [46] and extends beyond them, particularly by focusing on the likelihood of identifying the participants’ relevant expressed and felt needs.

To allow for deductive and inductive analyses and for a constant reflection of the researchers on their roles, we chose the method of reflexive thematic analysis. According to the handbook of Braun and Clarke, five steps were implemented: (i) familiarisation with the data, (ii) coding the data, (iii) generating initial themes, (iv) reviewing and developing themes, and (v) refining, defining and naming themes [47]. For this process, MAXQDA, Miro Board, and Microsoft Office Word were used as primary tools. In the results, all direct quotations have been anonymized and translated into English, are marked with citations, and provided with source references in brackets.

Quantitative data obtained from the standardized scales were analysed descriptively. Continuous variables were summarized using means and standard deviations (SD) or medians and measures of dispersion, as appropriate, and supplemented by ranges. Due to the small sample size, no inferential statistical analyses were performed. Scale scores were calculated and interpreted according to the respective scoring instructions and established reference or cut-off values.

The analysis was conducted by two researchers who previously moderated the focus groups. A female psychologist (SMH) analysed the results of the focus group and diaries of patients, and a female physician (AK) analysed the results of the parental focus groups. The researchers subsequently cross-validated their results, and a third researcher, a female social worker (ME) who did not participate in focus groups but was familiar with this method subsequently validated all results. The latter researcher and the physician were experienced in the diagnosis and treatment of ME/CFS and had been involved in prior clinical care for some study participants. Their interest in the topic arose from experiences in clinical context. They both had no experience in qualitative research. The psychologist had no practical experience with ME/CFS nor any contact with study participants prior to the start of the study. She was experienced in qualitative research, and her interest in the topic arose from prior research collaboration with the clinical team. None of the three researchers involved in the study had personal experiences with the disease nor any personal relationship with any study participants or other ME/CFS patients. All participants had received treatment at the study center, were informed about the respective roles of the researchers and their goal to implement a treatment model based on the results of the study.

After the analysis, the results were presented to interested participants with the opportunity to comment or criticize.

## Results

### Baseline characteristics of the samples

The baseline characteristics of the samples are shown in Table 1. In total, 32 families living in Bavaria were contacted, and 17 of them participated in the study. The number of participating members per family differed. Either the patient only, one or both parents or parents and their child took part.

**Table 1:** Baseline characteristics of the groups

	All	Adult Patients	Adolescent Patients
Patients	*N* = 14	*N* = 8	*N* = 6
Total number (*n*)	14	8	6
Age in years^1^	18.5 (3.75)	19.5 (3.25)	15.5 (1.0)
Females	10	7	3
Males (*n*)	4	1	3
Focus group method (*n*)	5	5	0
Diary method (*n*)	9	3	6
Residence in Bavaria	14	8	6
Functional status reduced to50–70% of normal^2,3^	7	N/A	N/A
Functional status reduced to< 50% of normal^2,3^	5	N/A	N/A
Functional status not reported^2,3^	2	N/A	N/A
Disease duration > 6months	14	8	6
Parents			
Total number (*n*)	18	8	10
Age in years (interquartile range)	52.0 (5.0)	56.5 (6.5)	52.0 (1.75)
Females (*n*)	13	7	6
Males (*n*)	5	1	4
Focus group method (*n*)	18	8	10
Residence in Bavaria	18	8	10

^1^Median (IQR)

^2^All frequencies below 5 were reported as N/A to reduce the risk of participant identification and to maintain confidentiality in the context of small cell sizes

^3^Detailed description in Fig. 1

Fourteen patients aged 12 to 24 years (median age 18.5 years) participated in the study. Of these, five adults (median age 19.5 years) participated in focus groups, but only two attended all sessions. At least three patients were present per focus group session. Nine patients used the diary method exclusively, and all of them completed the whole set of topics. Participants of the diary approach were aged 12 to 24 years (median age 16.0 years), including six children and adolescents (median age 15.5 years) and three adults (median age 23.0 years). All patients had a disease duration of at least six months.

Eighteen parents aged 42 to 61 years (median age 52.0 years) participated in focus groups, including 13 mothers and five fathers, with three couples participating either together or in separate sessions. In each session, the number of participating parents ranged between four and seven. Parents were divided into three focus groups: one including parents of adult patients only, one with parents of affected adolescents or children only, and one mixed group. The latter mix was necessary as the remaining number of parents was too small to generate two separate groups. The mixed group included two parents of adult patients and three parents of adolescent patients.

### Description of Study Population via Patient-Reported Outcome Measures (PROMs)

PROMs were only available from 12/14 (86%) participating patients and 17/18 (94%) parents. Two patients did not answer the questionnaires despite being reminded three times, possibly due to PEM. One parent did not answer despite three reminders. However, the spouse of the latter possibly indicated a misunderstanding of how many parents per family were invited to fill in the PROMs.

#### Bell score

**Fig. 1:**
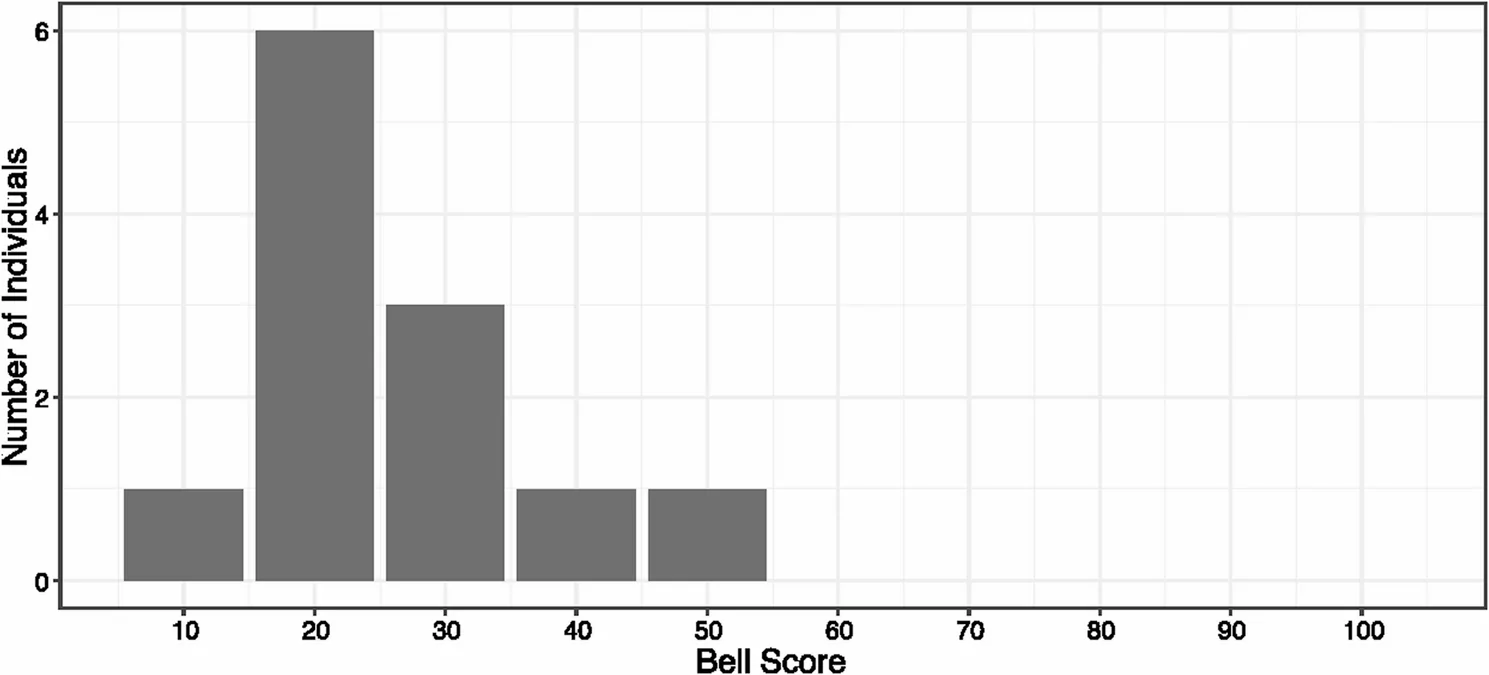
Bell scores of patients. Lower scores indicate increasing physical impairment, with 100 representing normal physical functioning. The majority of patients demonstrated substantial functional limitations

As illustrated in Fig. 1- Bell scores of patients, the median Bell scores of the 12 ME/CFS patients were very low, with 20 (IQR 10), ranging between 10 and 50 points. The Bell score of 20 points is described as follows: The patient has moderate to severe symptoms at rest and is unable to perform strenuous activity. Overall, activity levels are 30 to 50% of expected. The patient is unable to leave the house except rarely, is confined to bed most of the day, and is unable to concentrate for more than 1 h/day [37].

#### Short form 12 health survey questionnaire

**Fig. 2:**
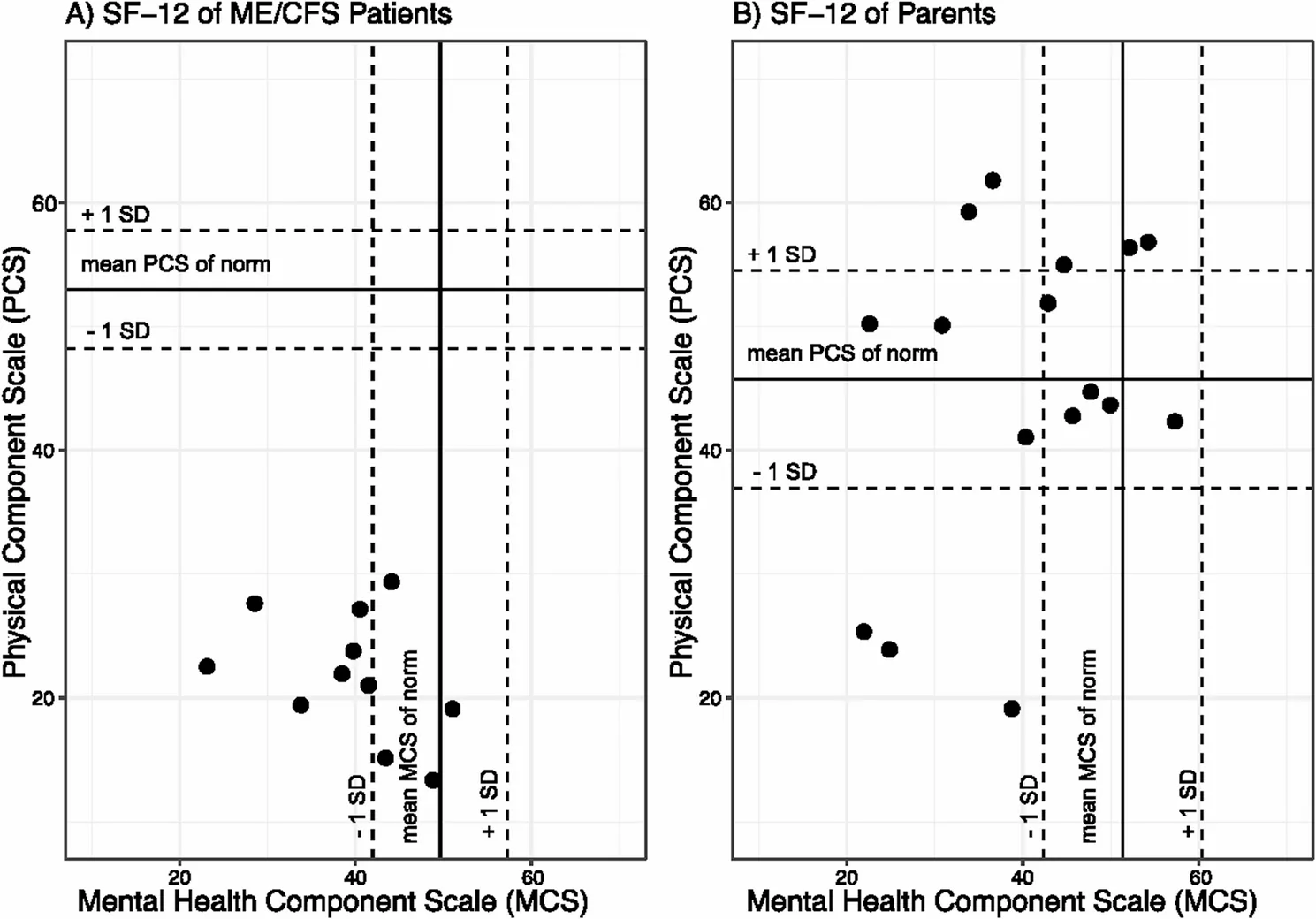
Short form-12 health survey questionnaire scores of patients (**A**) and parents (**B**). Dashed horizontal and vertical lines represent ± 1 standard deviation from the mean Physical Component Summary (PCS) and Mental Component Summary (MCS) scores of the German normative population (1998). The reference age ranges were 14–20 years for patients and 51–60 years for parents. Higher scores indicate better health status. In each group, two identical results are displayed as single dots located within the ± 1 SD range

In the patient group (*n* = 12), the PCS scores demonstrate substantial impairment, whereas the MCS scores were also reduced, but to a lesser extent. In the parental sample, MCS scores were reduced, whereas PCS scores were within the norm, as depicted in Fig. 2 - SF-12 of patients and parents.

#### Hospital anxiety and depression scale, german version

**Fig. 3:**
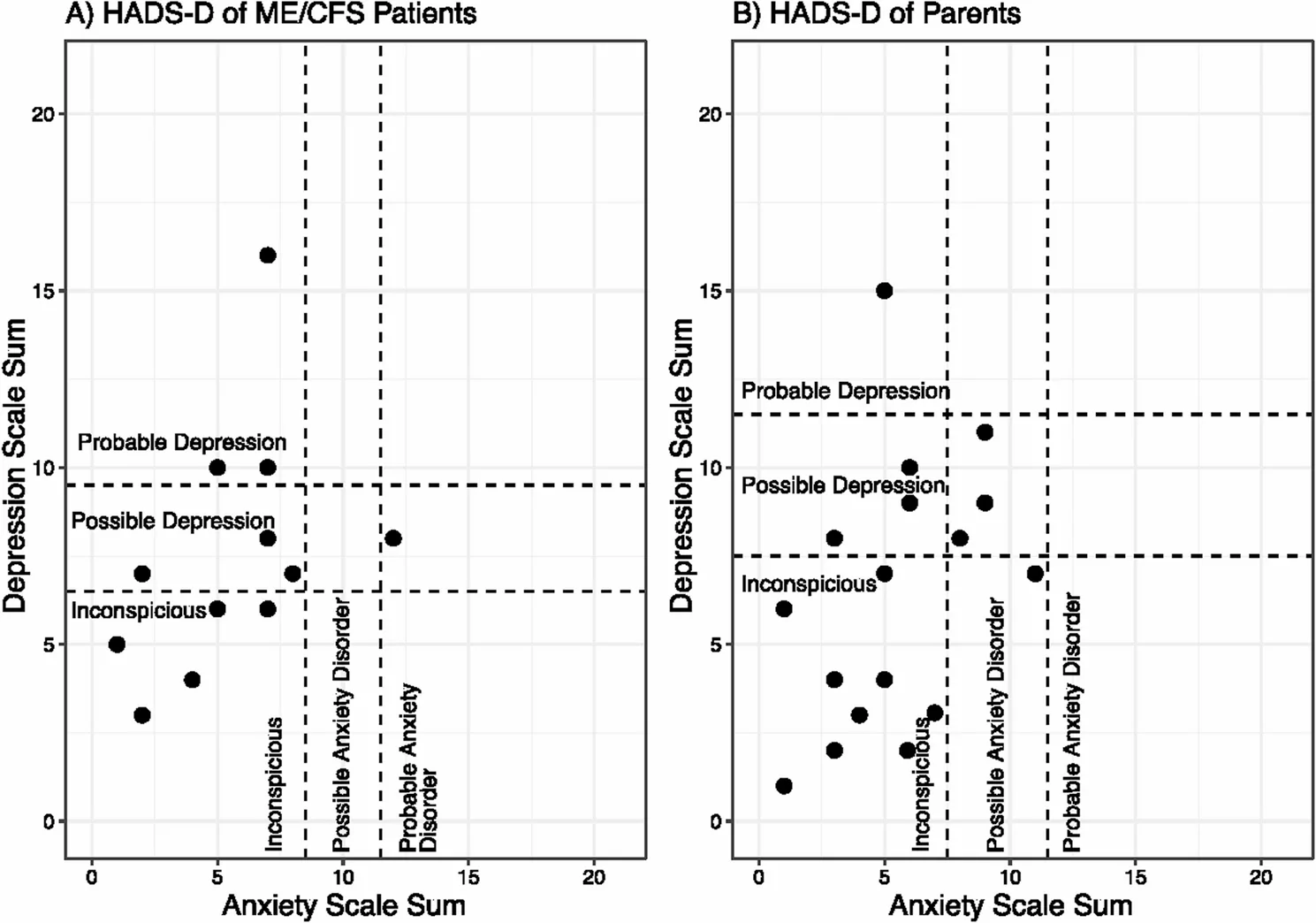
Hospital Anxiety and Depression Scale (HADS-D) scores for patients (**A**) and parents (**B**). Dashed lines represent threshold values. For adolescents (**A**), cut-offs are 8.5 and 11.5 on the anxiety scale, and 6.5 and 9.5 on the depression scale. For adults (**B**), the respective thresholds are 7.5 and 11.5. These cut-offs distinguish between normal, borderline, and clinically relevant symptom levels

Figure 3**- **HADS-D of patients and parents depicts the scores for depression and anxiety of ME/CFS patients and parents, respectively. Most participants in the ME/CFS sample showed scores in the norm for anxiety. However, one participant was found to have elevated scores for anxiety, being in the range that warrants urgent examination. Seven participants showed higher scores on the depression scale than their peers, with three being in the range for urgent examination. In the parental sample, four participants showed elevated scores on the anxiety scale, none in the critical range and seven participants showed elevated scores on the depression scale, with one being in the critical range.

### Qualitative results of focus groups and diary method

**Fig. 4:**
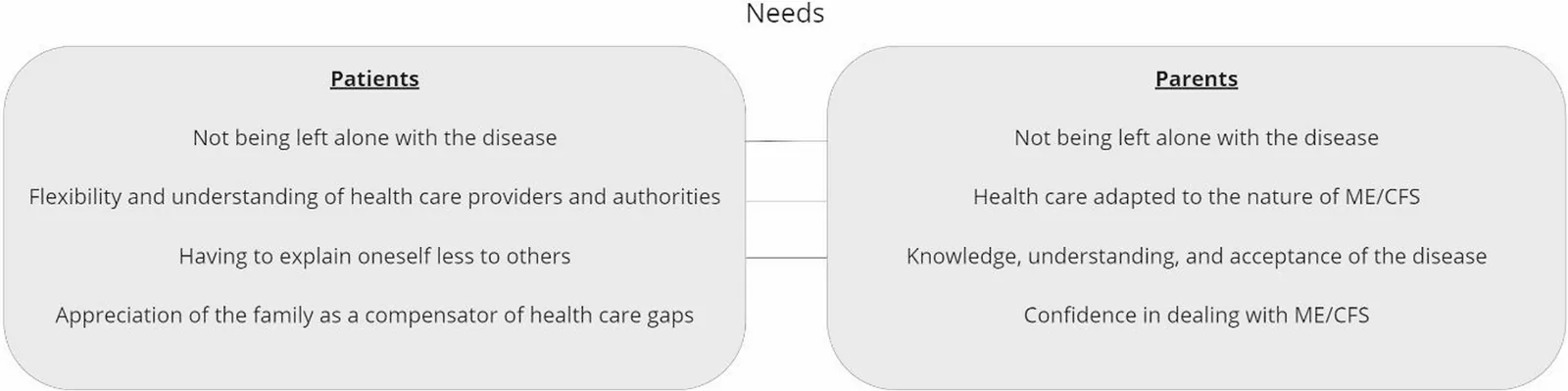
Identified needs from qualitative analysis. Four central themes emerged from discussions with ME/CFS patients (left). Four partially overlapping themes were identified in the parent group (right). Arrows indicate areas of conceptual overlap between patient and parent perspectives

In each sample, four needs were identified (Fig. 4 - Needs of patients and parents). All direct quotations have been anonymized and translated into English, are marked with citations, and provided with source references in brackets. In the following, these needs are being presented as themes and explained in more detail. In addition, the impact of the disease on the individuals with ME/CFS as well as on the whole family was analysed, which is presented as Supplementary Material 1 - Additional results.

### Qualitative results of the ME/CFS sample

Among the adolescents and young adults with ME/CFS, the following four main themes emerged.

#### Not being left alone with the disease

Participating patients reported that the diagnosis was usually delayed, and false differential diagnoses were attributed to the symptoms in some cases until ME/CFS was considered. This longstanding uncertainty about the symptoms led patients to a sense of being left alone. They, moreover, referred to the fact that only a few specialized health care centers exist and that the existing centrs are not accessible to all patients with ME/CFS, which further supported their feeling of being left alone with the disease. Young patients wished for low-threshold access to healthcare without exploiting all individual energy resources.

*“Easier accessibility and online office hours to save energy. Would like to have a “hotline” to get advice on my own.” (6.4 social contacts)*

The feeling of being alone also refers to social isolation. Many patients do not have enough energy resources to keep in contact with friends or to pursue leisure activities. The lack of understanding from others increases social isolation. Patients perceived contact with other individuals with present or prior ME/CFS as helpful since there was no need to explain oneself, since the feeling of being alone with this disabling condition was reduced, and since advice on how to deal with the symptoms could be exchanged between affected people. Young patients thought they could profit from patient interactions and wished for a communication platform organized by healthcare providers.

*“I am very grateful and happy about the exchange with the girls I was with in [rehabilitation]. I am still in contact with some of them*,* and the exchange with other affected people helps me.” (8.4 social contacts)*

*“It would help a lot to be able to talk about individual issues*,* such as school issues*,* to learn how others have overcome hurdles.” (0.4 social contacts)*

#### Flexibility and understanding of providers and authorities

The rigidity of existing care was mentioned as another issue. Many patients experienced a worsening of their condition due to outpatient visits, which could be adapted by personalized care.

*“One should be more cautious not to schedule too many appointments during medical visits/rehab. And listen more to how many sessions one is comfortable with - therapy should be highly individualized because of the risk of being overwhelmed.” (1.1 physical activity)*

Patients reported having good strategies to cope with limited energy resources, for example, “using the *read-aloud function on some websites” (0.4 Mental Activity)* when reading is impossible. However, they explained further that these only worked with a supporting system that allowed implementing their strategies. Patients asked for a higher level of empathy from the environment because only if there was any awareness of the individual needs of these patients and the possibility of implementing individualized care settings were patients able to use their strategies.

*“In case I am able to make it to a doctor’s office*,* I have to lie down right away. Unfortunately*,* not every private practice shows an understanding of this (need).” (8.1 physical activity)*

#### Having to explain oneself less to others

Patients with ME/CFS constantly feel like having to explain themselves to others. As they look healthy on the outside and since exhaustion usually becomes apparent with a time delay, patients described that they constantly must explain their disease and their specific needs even in their close environment. This need to explain oneself also becomes apparent when dealing with authorities to obtain support or communicating with health care staff.

*“Furthermore*,* I would wish for help and support in dealing with official matters. Having to explain myself and the disease over and over again and having to fight for everything is cognitively very exhausting.” (8.3 mental activity)*

Young people with ME/CFS also demand “*a better training of [general practitioners/paediatricians] in relation to ME/CFS” (1.1 Physical Activity)* to avoid the need for constant explanations, as it consumes energy reserves that either are not available or needed elsewhere.

#### Appreciation of the family as a compensator for health care gaps

In many cases, the family took over the entire care, including all disease-related organizational needs, and was often the only social contact for the patient.

*“If I didn’t have my mother, I would be totally lost…” (8.2 self-care)*

Young patients wished for support not only for themselves but also for their families to relieve their burden of care.

*“I would appreciate it*,* but not for me*,* rather to support my parents. And to reduce the extra work that I bring with me here in my situation.” (0.5 self-care)*

The lack of recognition of the severity of the disease and its effects on families might go along with insufficient appreciation of the family’s achievements. Patients perceive themselves as a burden to their families.

### Qualitative results of the parental sample

Among parents of ME/CFS patients, the following four themes emerged:

#### Not being left alone with the disease

Parents felt left alone, excluded from society, in their new demanding role as caretakers of a chronically ill child, and often confronted with resistance from their surroundings. Parents reported feeling specifically unsupported regarding the care for their child.

Parents wished for competent, easily available, and continuous medical support. Furthermore, parents stated that they felt left alone with bureaucratic issues and with fighting authorities for care and support.

*“A contact person for all these tasks regarding authorities*,* so that someone can take over all tasks in a bundle and thereby support the people. Everyone has to fight their way through individually*,* so it would be a tremendous help*,* and I think it would be better to proceed in a bundle than to have everyone fight for themself individually.” (O1*,*48)*

Parents asked for some relief for families, for example, through a visiting service. Moreover, parents wished for support in networking with other affected families and for a structured exchange to feel understood, to not be alone with the disease, and to profit from each other’s experiences.

*“For me*,* the exchange with other affected people was very helpful*,* […] when you don’t have to explain yourself for […] when someone says*,* I know exactly how you feel*,* I know it all.” (G2*,* 36)*

#### Health care adapted to the nature of ME/CFS

Parents reported that existing structures of care and support, in principle, might be useful for patients with ME/CFS, but most often were not sufficiently adapted to the specific needs of young patients with ME/CFS. The parents’ experiences indicated wide variation in the type and degree of support depending on the individual care provider, and that there was no systematic support for affected families. They explained that experienced support offers did not meet the needs of people with ME/CFS as a chronic disease – for example, long-term medical prescriptions or regular appointments over a long period of time.

*“There was the appointment at […]*,* which was so good*,* and then a process started*,* our child got worse*,* and we stood next to it and actually had no one to simply ask*,* share our concerns with*,* discuss what was actually happening here.” (G2*,*52)*

Parents stated that high flexibility during patient care was necessary since the course of the disease, as well as the daily health condition of patients, were unpredictable. They explained further that patients might not be able to attend an outpatient visit or make a phone call. Many parents asked for easier accessible care, such as home care or telemedicine:

“*We found a psychologist who was flexible enough to offer consultations by phone and […] also flexible enough to accept our cancelling of the appointment fifteen minutes before it started when it was just not possible that day.” (O1*,*44)*

*“The disease calls for home care because every attempt to leave the house is incredibly exhausting*,* and in our case*,* it always resulted in a relapse. Visiting the doctor’s private practice once means facing a total crash for two weeks. Thus*,* rather via telemedicine*,* e.g. video session*,* or via home visit.” (G1*,*101)*

#### Knowledge, understanding, and acceptance of ME/CFS

A central aspect during the discussions with parents was the need to feel understood. Parents *“have to explain [themselves] all the time. And this constant explaining is a great effort […]” (G1*,*90).* They reported that they had to educate their environment regarding the type of disease as well as medical and psychosocial needs, and to defend their children and themselves against misconceptions or prejudices. They felt that the disease was not only unknown in the general society but also among healthcare professionals.

*“It is astonishing that there are some [physicians] who still don’t know the disease at all.” (G2*,*13)*

Furthermore, parents made the experience that even if health professionals had heard about the disease, they had not familiarized themselves with it or did not accept it.

*“So*,* you often get shrugs of the shoulders*,* I don’t know anything about that and I don’t want to deal with it and that’s why there are no medical certificates*,* so that’s wiped off the table relatively quickly.” (O1*,* 20)*

Parents often felt themselves or their children judged or stigmatized by health care professionals. They explained that they were frequently not taken seriously when describing complaints or found themselves accused of not caring for their child appropriately. Parents, moreover, reported that the lack of knowledge among healthcare providers often leads to a delay in diagnosis or inadequate treatment, which might cause a significant worsening of the disease.

*“I think it is a tragedy that our daughter would not be at this point if we had not had the wrong advice from doctors from the beginning. If the doctors had been educated at the time she had COVID and had not recommended constant sports therapy*,* she would be in a very different health condition than she is now…” (B1*,*63)*

Parents asked for more research and appropriate education of health care professionals and authorities to guarantee appropriate support for patients with ME/CFS.

*“So*,* for me the absolutely most important point is: Education of primary care physicians or the first institution you meet with this problem.” (B1*,*63)*

#### Confidence in dealing with ME/CFS

Parents pointed out that sometimes they felt helpless and insecure about how to deal with the disease and their affected child. When first confronted with the disease, they point out that an understanding of the disease and its specific characteristics has to be developed.

“*For us*,* the biggest challenge in the beginning was coming to terms with it ourselves*,* how to communicate it*,* how to deal with it.” (B2*,*3)*

In addition, parents state that it is necessary to deal with the disease and the changes that result for the whole family. The data show that dealing with the affected child is difficult in multiple ways, especially since most children had been completely healthy before the onset of ME/CFS and now had to change from their developing autonomy back to total dependency on their families. A challenging question for parents was how to avoid PEM while motivating their child to work on improvements and social participation.

*“At the beginning of the disease*,* I always found it difficult to balance the two: How much to slow down and how much to try out anyway. So yes*,* pacing*,* yes*,* considering the limited strength*,* but yet not being afraid to try out alternative solutions.” (G2*,*11)*

Parents added that not only parents but also siblings and other family members were struggling with these issues, their new role, and their interaction with the affected family member. Many parents shared worries about the future of their affected children. They explained that planning their future was complicated by the uncertainties about the course of the disease and options for treatment. The topics of school and education were of very high priority during these discussions.

*“Currently*,* she is most likely aiming for a distance learning program*,* probably part-time. And*,* of course*,* it’s difficult to find the right address. Then*,* of course*,* you don’t get BAföG*^1^*if it’s part-time*,* and so on. So*,* all in all*,* it’s what we’re most concerned about now because it also has a lot to do with future perspectives*,* which*,* especially with the disease and especially with young people*,* play a major role.” (G1*,*3)*

Parents asked for comprehensive information about the disease and for adequately processed updates about ME/CFS research achievements. Furthermore, parents wished to be more intensively involved in the treatment *“so that parents could also speak with the doctors on-site” (O2*,*54)* and to receive personal counselling regarding optimal support by families.

## Discussion

This study used qualitative methods to assess the needs of severely affected young people with ME/CFS and their families, thereby gathering information that could help design appropriate future care models. The four needs found in each group were (i) Not being left alone with the disease (identical in both groups), (ii) Flexibility and understanding of providers and authorities (Patients) / Health care adapted to the nature of ME/CFS (Parents), (iii) Having to explain oneself less to others (Patients) / Knowledge, understanding, and acceptance of ME/CFS (Parents), and (iv) Appreciation of the family as a compensator for health care gaps (Patients) / Confidence in dealing with ME/CFS (Parents).

Although the data were collected from patients and parents independently, and analysed by two different researchers using different techniques, the results overlapped remarkably, which demonstrates the urgency of the four needs found.

The most prominent need was identical in both groups: affected adolescents and young adults, as well as their parents, felt abandoned. For patients, this feeling resulted from both long journeys to diagnosis, as reported in the literature [22] and social isolation caused by the disease. Patients, therefore, looked for strategies to maintain social participation and education by fighting for minimal school attendance [22]. Parents also felt socially isolated, but their feelings related to being left alone with the care for their affected child. They were not primarily looking for support for themselves but to be able to care for their child in the best possible way. These results are in line with findings from other studies in which parents indicated that they care blindly without guidance and support [35], and felt like outsiders in society [48]. Both the removal of barriers to adequate care, as well as providing contact to other affected families, might improve this situation.

Second, ME/CFS families wished for more flexibility and better understanding by health professionals to support appropriate self-management. In contrast to previous literature [22], our patient sample struggled not so much with being guided in pacing and coping strategies but rather with getting insufficient support for consistent implementation of these tasks during physician visits. The fact that the study sample had already successfully implemented these strategies at home was most likely due to the fact that they were well-instructed at the time of diagnosis and had been self-managing before this study. Patients expressed a preference for more individualized treatment approaches rather than rigid care structures, which were often perceived as being associated with a worsening of their health status. In addition, participants highlighted the need for greater empathy and a more patient-centred recognition of their individual needs. Parents asked for a medical system adapted to the unique features of the disease to prevent their child’s health from deteriorating due to inappropriate inpatient or outpatient facilities. In the current ME/CFS literature, assessments of caregiver needs are rare. However, its importance is unequivocal and adapted care models have shown promising results in multiple studies [20, 49]. The adaptation of our study proves the need for an adapted care structure that allows flexibility to be essential. Since repetitive online sessions were too strenuous for the majority of the patients, we alternatively offered an adapted diary method, which was well received by most patients and offered the necessary flexibility and individuality, which was found to be one of the major needs.

The third theme was also similar for both samples. ME/CFS patients reported that they often had to explain themselves and their disease to those around them – even their closest family and friends – and that this was very exhausting. In particular, explaining themselves to health care providers and authorities to obtain support was cited as a significant burden for ME/CFS patients in view of the limited energy resources. The sample of parents felt that they had to explain their child’s disease and the limitations that came with it, and had to constantly defend their own actions regarding the illness. This aligns with previous studies indicating that parents are confronted with a lack of medical knowledge, understanding and even rejection from those around them, including medical staff [50]. The lack of awareness in the medical field and the general population mentioned by the participants is a major obstacle to a correct and timely ME/CFS diagnosis as well as to appropriate treatment and education, which is often associated with stigma and isolation [34, 50].

As a fourth theme, parents were concerned about how to properly deal with the child’s situation and indicated that they needed help with this. This was consistent with previous studies that pointed to uncertainties related to finding a correct diagnosis, addressing medical ambiguities about the disease and appropriate care, estimating prognosis [48] and caring for their child, especially in the context of non-adult patients [35]. Confidence in these matters might be achieved by involving parents in specialized patient education programs, indicating specialized healthcare facilities, and continuous follow-up by specialized medical professionals. High levels of psychological distress in parents were frequently reported in the ME/CFS literature [17] and were also evident in our focus groups. However, it was not articulated as a need for support from our parent sample. Despite their high level of stress, parents would rather seek advice on how to deal with their child’s situation than ask for direct support for themselves.

Notably, patients emphasized the importance of appreciating the family’s and one’s own efforts to cope with the disease and with the lack of medical care. In line with published evidence, some patients felt guilty for the burden on the family caused by the disease and the resulting changes in the family’s life [15]. Patients expressed a need for additional support in coping with their illness. Remarkably, their concerns focused less on their own support needs and more on the well-being of their families, highlighting their awareness of the considerable burden that the disease places on caregiving family members.

The PROMs indicated reduced health-related quality of life in both samples compared with population norms. This demonstrates the high burden parents are under. In contrast to previous studies with homebound adult ME/CFS patients that demonstrated both high levels of psychological and physical distress using PROMs [5], we found that our patients were less impaired in terms of psychological versus physical well-being. The latter might be multifactorial. Young patients may benefit from parental support, fear being “psychologized”, and might tend to express psychological distress through physical symptoms. The importance of including family members in the needs assessment of young people with ME/CFS was demonstrated here by the findings that some patients were unable to participate in the evaluation due to limited energy resources. The involvement of relatives thus made it possible to give a voice to very severely affected ME/CFS patients and to get insight into the enormous effort of parents to properly manage the disease, support their affected child, and fill gaps in the current healthcare system.

### Strengths and limitations

The results of the study have to be viewed in context of the researchers’ experiences. Having the experience of working with patients affected by the disease may have helped the researchers to understand the expressed needs. On the other hand, there might have been bias due to the researchers’ own needs in clinical practice. As two of the researches knew some participants prior to study begin, the personal experiences with these participants in the clinical context may have impacted the analysis of the expressed needs in the study. It may have also had an impact on the group dynamic, in which some participants knew the researchers, while others did not. As all participants or their relatives had received treatment at the study center prior to the study, it may have influenced their willingness to participate as well as their trust in the researchers and willingness to express needs openly. Also, a possible restraint from critics of the health system may have resulted from the relationship.

To the best of our knowledge, this study represents the first exploration of needs of severely affected young ME/CFS patients in Germany, including the parents’ perspectives. The qualitative evaluation approach allowed for the identification of several dominant needs of patients and parents. The introduction of the diary method into the qualitative analyses underscored the potential of innovative research methods in the context of severe ME/CFS in young people. The positive feedback of the participants, such as “being heard”, underlined the success of the methodological approach. Moreover, both parents and patients stated that they benefited from the exchange with other families in focus groups, indicating that group settings might be a promising strategy in future healthcare models for ME/CFS in young people.

As a limitation, the number of focus groups with ME/CFS patients was small. However, we included additional patients via the diary method and suggest combining these methods in future studies. In both groups – patients and parents – data saturation was reached in spite of the small sample size. Second, since all families lived in Bavaria and were treated at a single Bavarian tertiary care center their needs might not be representative; additional or other needs might be more prominent in other regions. Third, the needs of the addressed families might not be fully reflected by the perspectives of patients and parents only.

### Implications for research and practice

Future studies should thus also address the needs of siblings and other household members. Comparing the needs of affected families with the needs of local healthcare providers and school staff might additionally contribute to elucidating gaps in care.

Taken together, this study identified the dominant needs of young people with severe ME/CFS and their parents and thereby indicated several targets for intervention. Addressing these targets, such as those aimed at in the Post-COVID Kids Bavaria and BAYNET FOR ME/CFS projects, is expected to contribute to improved care for young people with ME/CFS and to support their parents.

## Conclusion

This study illustrates the unmet needs pf paediatric and young adult ME/CFS patients and their caregiving parents. Participants underscore the importance of receiving support in order not to feel left alone with their condition. They emphasized on an adapted and flexible treatment. Patients and parents additionally demonstrated the significance of increasing knowledge, understanding and acceptance of ME/CFS among health care professionals as well as in society. Finally, parents wished to be more integrated in the treatment process to gain confidence in dealing with the disease, whereas patients emphasized the importance of an appreciation of their families’ caregiving work and support for their families in order to reduce the burden of the disease for the whole family.

The findings highlight the relevance of more individualized and family-centred approaches to care. Addressing these needs may contribute to improved healthcare experiences and quality of life for affected patients and their families.

## Supplementary Information

Supplementary Material 1: Additional Results

Supplementary Material 2: Sociodemographic data of the parental sample.

## Data Availability

All original data of this study are stored on an external storage device stored in the children’s hospital. To protect study participant privacy, only the authors Alissa Kircher, Sandra Mayer-Huber and Maria Eberhartinger have access to stored original data of this study. No hyperlink is available.

## Footnotes

1 Government funding for students according to the Bundesausbildungsförderungsgesetz (BAföG), a German Federal Training Assistance Act.

## References

[1] Bakken IJ, Tveito K, Gunnes N, et al. Two age peaks in the incidence of chronic fatigue syndrome/myalgic encephalomyelitis: a population-based registry study from Norway 2008–2012. BMC Med. 2014;12:167. 10.1186/s12916-014-0167-5. PMC4189623 25274261

[2] Institut für Qualität und Wirtschaftlichkeit im Gesundheitswesen. Myalgische Enzephalomyelitis / Chronic Fatigue Syndrome (ME/CFS): Aktueller Kenntnisstand; Abschlussbericht. Köln. IQWiG-Berichte. https://nbn-resolving.org/urn:nbn:de:101:1-2023062608450760557308.

[3] Jason LA, Katz BZ, Sunnquist M, Torres C, Cotler J, Bhatia S. The Prevalence of Pediatric Myalgic Encephalomyelitis/Chronic Fatigue Syndrome in a Community–Based Sample. Child Youth Care Forum. 2020;49(4):563–579. 10.1007/s10566-019-09543-3. PMC8186295 34113066

[4] Clayton EW. Beyond myalgic encephalomyelitis/chronic fatigue syndrome: an IOM report on redefining an illness. JAMA. 2015;313(11):1101–1102. 10.1001/jama.2015.1346. 25668027

[5] Pendergrast T, Brown A, Sunnquist M, et al. Housebound versus nonhousebound patients with myalgic encephalomyelitis and chronic fatigue syndrome. Chronic Illn. 2016;12(4):292–307. 10.1177/1742395316644770. PMC5464362 27127189

[6] Strassheim V, Newton JL, Collins T. Experiences of Living with Severe Chronic Fatigue Syndrome/Myalgic Encephalomyelitis. Healthcare (Basel). 2021;9(2):168. 10.3390/healthcare9020168. PMC7914910 33562474

[7] Carruthers BM, Jain AK, Meirleir KL de, et al. Myalgic Encephalomyelitis/Chronic Fatigue Syndrome. Journal of Chronic Fatigue Syndrome. 2003;11(1):7–115. 10.1300/J092v11n01_02.

[8] Hwang J-H, Lee J-S, Oh H-M, Lee E-J, Lim E-J, Son C-G. Evaluation of viral infection as an etiology of ME/CFS: a systematic review and meta-analysis. J Transl Med. 2023;21(1):763. 10.1186/s12967-023-04635-0. PMC10612276 37898798

[9] Peo L-C, Wiehler K, Paulick J, et al. Pediatric and adult patients with ME/CFS following COVID-19: A structured approach to diagnosis using the Munich Berlin Symptom Questionnaire (MBSQ). Eur J Pediatr. 2023. 10.1007/s00431-023-05351-z. PMC10951047 38095713

[10] Pricoco R, Meidel P, Hofberger T, et al. One-year follow-up of young people with ME/CFS following infectious mononucleosis by Epstein-Barr virus. Front Pediatr. 2023;11:1266738. 10.3389/fped.2023.1266738. PMC10830704 38304441

[11] Rowe KS. Long Term Follow up of Young People With Chronic Fatigue Syndrome Attending a Pediatric Outpatient Service. Front Pediatr. 2019;7. 10.3389/fped.2019.00021. PMC6393360 30847333

[12] Ghali A, Lacout C, Fortrat J-O, Depres K, Ghali M, Lavigne C. Factors Influencing the Prognosis of Patients with Myalgic Encephalomyelitis/Chronic Fatigue Syndrome. Diagnostics (Basel). 2022;12(10). 10.3390/diagnostics12102540. PMC9600584 36292229

[13] Winger A, Kvarstein G, Wyller VB, et al. Health related quality of life in adolescents with chronic fatigue syndrome: a cross-sectional study. Health Qual Life Outcomes. 2015;13(1). 10.1186/s12955-015-0288-3. PMC4490669 26138694

[14] Froehlich L, Hattesohl DB, Cotler J, Jason LA, Scheibenbogen C, Behrends U. Causal attributions and perceived stigma for myalgic encephalomyelitis/chronic fatigue syndrome. J Health Psychol. 2022;27(10):2291–2304. 10.1177/13591053211027631. PMC9434257 34240650

[15] Rowe PC, Underhill RA, Friedman KJ, et al. Myalgic Encephalomyelitis/Chronic Fatigue Syndrome Diagnosis and Management in Young People: A Primer. Front Pediatr. 2017;5:121. 10.3389/fped.2017.00121. PMC5474682 28674681

[16] Collin SM, Crawley E, May MT, Sterne JAC, Hollingworth W. The impact of CFS/ME on employment and productivity in the UK: a cross-sectional study based on the CFS/ME national outcomes database. BMC Health Serv Res. 2011;11:217. 10.1186/1472-6963-11-217. PMC3184626 21923897

[17] Missen A, Hollingworth W, Eaton N, Crawley E. The financial and psychological impacts on mothers of children with chronic fatigue syndrome (CFS/ME). Child Care Health Dev. 2012;38(4):505–512. 10.1111/j.1365-2214.2011.01298.x. 21880054

[18] McManimen SL, Devendorf AR, Brown AA, Moore BC, Moore JH, Jason LA. Mortality in Patients with Myalgic Encephalomyelitis and Chronic Fatigue Syndrome. Fatigue. 2016;4(4):195–207. 10.1080/21641846.2016.1236588. PMC5218818 28070451

[19] James Daniell, Johannes Brand, Dirk Paessler, Joerg Heydecke, Simon Schoening, Amy Mclennan. The rising cost of Long COVID and ME/CFS in Germany; 2025.

[20] Montoya J, Dowell T, Mooney A, Dimmock M, Chu L. Caring for the Patient with Severe or Very Severe Myalgic Encephalomyelitis/Chronic Fatigue Syndrome. Healthcare (Basel). 2021;9(10):1331. 10.3390/healthcare9101331. PMC8544443 34683011

[21] Hannon K, Peters S, Fisher L, et al. Developing resources to support the diagnosis and management of Chronic Fatigue Syndrome/Myalgic Encephalitis (CFS/ME) in primary care: a qualitative study. BMC Fam Pract. 2012;13:93. 10.1186/1471-2296-13-93. PMC3573921 22998151

[22] Drachler MdL, Leite JCdC, Hooper L, et al. The expressed needs of people with chronic fatigue syndrome/myalgic encephalomyelitis: a systematic review. BMC Public Health. 2009;9:458. 10.1186/1471-2458-9-458. PMC2799412 20003363

[23] Ashby B, Wright B, Jordan J. Chronic Fatigue Syndrome: An Evaluation of a Community Based Management Programme for Adolescents and their Families. Child Adolesc Ment Health. 2006;11(1):13–18. 10.1111/j.1475-3588.2005.00383.x. 32811062

[24] Roche J, Tucker S. Extending the Social Exclusion Debate. Childhood. 2003;10(4):439–456. 10.1177/0907568203104004.

[25] Richards J, Chaplin R, Starkey C, Turk J. Illness Beliefs in Chronic Fatigue Syndrome: A Study Involving Affected Adolescents and their Parents. Child Adolesc Ment Health. 2006;11(4):198–203. 10.1111/j.1475-3588.2006.00409.x. 32810979

[26] Rangel L, Garralda ME, Levin M, Roberts H. The course of severe chronic fatigue syndrome in childhood. J R Soc Med. 2000;93(3):129–134. 10.1177/014107680009300306. PMC1297949 10741312

[27] Thornton EJ, Hayes LD, Goodwin DS, Sculthorpe N, Prior Y, Sanal-Hayes NEM. Managing Energy, and Shaping Care: Insights from Adults with Myalgic Encephalomyelitis/Chronic Fatigue Syndrome Through Co-Production Workshops. Am J Med. 2025;138(6):1001–1009. 10.1016/j.amjmed.2025.02.008. 39961545

[28] Parslow R, Patel A, Beasant L, Haywood K, Johnson D, Crawley E. What matters to children with CFS/ME? A conceptual model as the first stage in developing a PROM. Arch Dis Child. 2015;100(12):1141–1147. 10.1136/archdischild-2015-308831. PMC4680202 26453575

[29] Parslow RM, Anderson N, Byrne D, Shaw A, Haywood KL, Crawley E. Adolescent’s descriptions of fatigue, fluctuation and payback in chronic fatigue syndrome/myalgic encephalopathy (CFS/ME): interviews with adolescents and parents. BMJ Paediatr Open. 2018;2(1):e000281. 10.1136/bmjpo-2018-000281. PMC6307594 30613800

[30] Parslow RM, Harris S, Broughton J, et al. Children’s experiences of chronic fatigue syndrome/myalgic encephalomyelitis (CFS/ME): a systematic review and meta-ethnography of qualitative studies. BMJ Open. 2017;7(1):e012633. 10.1136/bmjopen-2016-012633. PMC5253584 28087544

[31] Sommerfelt K, Schei T, Angelsen A. Severe and Very Severe Myalgic Encephalopathy/Chronic Fatigue Syndrome ME/CFS in Norway: Symptom Burden and Access to Care. J Clin Med. 2023;12(4). 10.3390/jcm12041487. PMC9963221 36836022

[32] Harris K, Band RJ, Cooper H, Macintyre VG, Mejia A, Wearden AJ. Distress in significant others of patients with chronic fatigue syndrome: A systematic review of the literature. Br J Health Psychol. 2016;21(4):881–893. 10.1111/bjhp.12202. 27255790

[33] Sabes-Figuera R, McCrone P, Hurley M, King M, Donaldson AN, Ridsdale L. The hidden cost of chronic fatigue to patients and their families. BMC Health Serv Res. 2010;10:56. 10.1186/1472-6963-10-56. PMC2845126 20202216

[34] Webb CM, Collin SM, Deave T, Haig-Ferguson A, Spatz A, Crawley E. What stops children with a chronic illness accessing health care: a mixed methods study in children with Chronic Fatigue Syndrome/Myalgic Encephalomyelitis (CFS/ME). BMC Health Serv Res. 2011;11:308. 10.1186/1472-6963-11-308. PMC3228771 22078101

[35] Baken DM, Ross KJ, Hodges LD, Batten L. Experiences of carers of youth, adult children and spouses with ME/CFS. Chronic Illn. 2023;19(4):719–729. 10.1177/17423953221121696. 36259126

[36] Jason LA, Jordan K, Miike T, et al. A Pediatric Case Definition for Myalgic Encephalomyelitis and Chronic Fatigue Syndrome. Journal of Chronic Fatigue Syndrome. 2006;13(2–3):1–44. 10.1300/J092v13n02_01.

[37] Bell DS. Chronic Fatigue / Immune Dysfunction Syndrome: CFIDS, the disease of a thousand names. Lyndonville, N.Y.: Pollard Pub; 1991.

[38] Bell DS. The doctor’s guide to chronic fatigue syndrome: Understanding, treating, and living with CFIDS. Reading, Mass.: Perseus Books; 1995.

[39] Morfeld M, Kirchberger I, Bullinger M. SF-36: Deutsche Version des Short form-36 health survey: Manual. Göttingen, Bern, Wien, Paris: Oxford, Prag, Toronto, Cambridge, Ma, Amsterdam, Kopenhagen, Stockholm: Hogrefe. 2011.

[40] Bellach B-M, Ellert U, Radoschewski M. Der SF-36 im Bundes-Gesundheitssurvey. Bundesgesundheitsblatt - Gesundheitsforschung - Gesundheitsschutz. 2000;43(3):210–216. 10.1007/s001030050036.

[41] Herrmann-Lingen C, Snaith P, Zigmond AS, eds. Hospital anxiety and depression scale: HADS-D; deutsche Version; ein Fragebogen zur Erfassung von Angst und Depressivität in der somatischen Medizin; Testdokumentation und Handanweisung. 2. Aufl. Bern: Huber; 2005.

[42] White D, Leach C, Sims R, Atkinson M, Cottrell D. Validation of the Hospital Anxiety and Depression Scale for use with adolescents. Br J Psychiatry. 1999;175(5):452–454. 10.1192/bjp.175.5.452. 10789277

[43] Flynn R, Walton S, Scott SD. Engaging children and families in pediatric Health Research: a scoping review. Res Involv Engagem. 2019;5:32. 10.1186/s40900-019-0168-9. PMC6827239 31700676

[44] Maslow AH. A theory of human motivation. Psychological Review. 1943;50(4):370–396. 10.1037/h0054346.

[45] Wright J, Williams R, Wilkinson JR. Development and importance of health needs assessment. BMJ. 1998;316(7140):1310–1313. 10.1136/bmj.316.7140.1310. PMC1113037 9554906

[46] Denz-Penhey H, Murdoch JC. Service delivery for people with chronic fatigue syndrome: a pilot action research study. Fam Pract. 1993;10(1):14–18. 10.1093/fampra/10.1.14. 8477887

[47] Braun V, Clarke V. Thematic analysis: A practical guide. Los Angeles: SAGE; 2022.

[48] Mihelicova M, Siegel Z, Evans M, Brown A, Jason L. Caring for people with severe myalgic encephalomyelitis: An interpretative phenomenological analysis of parents’ experiences. J Health Psychol. 2016;21(12):2824–2837. 10.1177/1359105315587137. PMC4675701 26063209

[49] Kingdon C, Giotas D, Nacul L, Lacerda E. Health Care Responsibility and Compassion-Visiting the Housebound Patient Severely Affected by ME/CFS. Healthcare (Basel). 2020;8(3). 10.3390/healthcare8030197. PMC7551603 32635535

[50] Pheby DFH, Araja D, Berkis U, et al. A Literature Review of GP Knowledge and Understanding of ME/CFS: A Report from the Socioeconomic Working Group of the European Network on ME/CFS (EUROMENE). Medicina (Kaunas). 2020;57(1). 10.3390/medicina57010007. PMC7823627 33374291

