# Supplementary material for: Which needs do children, adolescents, and young adults with ME/CFS and their parents express and feel? - Qualitative study in Germany
Source: BMC Public Health. 2026 Sep 29;26:2942. doi: 10.1186/s12889-026-29549-6 (PMC13656452; doi:10.1186/s12889-026-29549-6)
Supplement: Supplementary file 1 [file 12889_2026_29549_MOESM1_ESM.pdf]

Supplementary Material 1: Additional Results

(A) Impact of the disease on the severely affected ME/CFS cohort using the International Classification of Functionality, Disability and Health (ICF)

ICF Component	Codes	Original citation
Body functioning and structures	*Fluctuation of symptoms*	*Well, I have days when I can't get out of bed at all. And then I have days when things somehow go a little better. (0.1 Physical activity)*
	Cognitive impairments	*Unfortunately, I forget the content quickly. Inability to concentrate. Brain fog with pain and forgetfulness prevent me from reading and retaining learning material. (1.4 Mental Activity).*
	Functional limitations after exertion	*My ability to concentrate usually remains the same throughout the day, only when I exert myself mentally or physically too much, I lose the ability to concentrate. (1.4 Mental activity)*
Environmental factors and personal factors	*Support factor parents*	*Since I live at home - thank God - I don't have to shop and clean the house. I wouldn't be able to do that at the moment. (5.4 Self-care)*
	Support factor physiotherapy	*Important for me are once a week physiotherapy on the machine and on the other hand once a week massage which do me a lot of good. Both of these appointments are very important for me during the week, because they are fixed and therefore an opportunity for me to get out. (4.4 Physical activity)*
	Support factor wheelchair	*Since I got the wheelchair, I can at least go shopping again. That was no longer possible before. On vacation, I can go on longer trips (up to one and a half hours) which would not be possible on foot. (7.3 Physical activity)*
	Barrier transportation services	*The most burdensome limitation for me today is definitely the lack of independence in terms of mobility. Since getting around by public transport still costs me a lot of energy, I am actually always dependent on someone to drive me, which limits me especially in terms of spontaneity. (3.4 Self-care)*
	Barrier Open space planning services	*If I could sit down more often, that would be very helpful for me. (6.1 Physical Activity)*
	Knowing your own limits	*I would say I know my limits, but sometimes it feels still like standing in front of a wall and I don't know where the wall is coming from. (0.1 Physical Activity)*
	Accepting a low degree of independence	*For me it was difficult from the beginning, that I was not able to do anything independently. (4.3 Self-sufficiency)*
Activities and Participation	*Standing and walking*	*I can walk a maximum of 5-10 minutes a day without feeling serious consequences. After a doctor's or any other appointment, I first need a few days of rest. I can't stand up for more than about 2 minutes, otherwise I get dizzy. If I walk a little longer, my legs start to give way and I have to lie down. (1.1 Physical activity)*
	Body care	*Showering is fine, but washing my hair is a real act. Rinsing it with water is okay, but washing hair is only possible on days when no other activities take place. (0.5 self-care)*

	Media consumption	*When I read a book, I don't even remember what I read. Watching TV is only possible at low volume and watching a movie is only possible with pauses. (4.1 Mental activity)*
	Contact with healthy peers	*It is just difficult to maintain many friendships via WhatsApp. Therefore, better fewer, but closer friends (that you also know for a longer time). (0.3 social contacts)*
	Community Life	*And I don't have that many other contacts and I don't meet new people as I'm not a part of social life anymore. (0.1 Physical activity)*

(B) Impact of the child's disease on the parental cohort using thematic analysis

Themes	*Codes*	*Original citation*
Reshaped family life	*Adapting family life to illness*	*With MECFS, normal family life as it used to be is no longer possible. (O1,24)*
	Less space for siblings	*It also has an immense impact on the siblings, they have to show a lot of consideration. (O1,24)*
	Professional cutback of parents	*Another factor: My wife has stopped working in order to take care of the child, as of course she cannot take care of herself alone. (O1,27)*
	Parents' social withdrawal	*I have actually lost friends because of this, because there was no understanding despite educational measures. (B2,8)*
Changed family relationships	*Dependence of the affected child on the family*	*He can't really move around independently outside the house anymore, so he is extremely dependent on us, which I see as problematic. (G1,9)*
	Promotion of autonomy versus necessary care	*What I notice as well is the challenge to me to pay attention to everything where I can promote his autonomy [...] to ask constantly - these things have to be done, what can you manage on your own? (G2, 28)*
	Change in the parents' partnership	*Also our partnership, we thought that now, as the children are growing up, we would march back into couple life, but that has changed completely. (G1,32)*
	Family division	*It took us a really long time to find a way how to deal with it, and unfortunately the only solution was to split up the family a bit. (B2,20)*
	Growing family ties	*I actually think that the family bond improved in a very positive way as well. (G2,27)*
Psychological stress of parents	*Helplessness and insecurity*	*It's not easy to handle, and I am not sure if we gave up too early, the question is how can we encourage someone to do a bit more and not fall into state of nothingness. (B1,2)*
	Identification with the child's suffering and guilty consciousness	*When you leave home as a mother or father, you feel guilty and think, how much I would like to stay at home instead of my daughter. (O1,26)*
	Parents' grief regarding the change in their own lives	*It is also the mourning for one's own life, because we all probably imagined it differently when the children slowly grow up and leave home. (O1,30)*
	Fears about the future	*She doesn't have a school-leaving certificate, and if things don't get better, she might not be able to get*

		one. We are not very old right now, but the question is, if we are no longer there, what will the situation be like, how can she lead her life then? (O1,36)
	Exhaustion due to constant fighting	*I only realize now, that the energy is gone to fight about anything else with authorities. (G2,30)*
	Little room for parents' own psychological stress	*I also have to ignore it a bit, otherwise I wouldn't be able to do anything at all (O1,30)*
