# Supplementary material for: Which needs do children, adolescents, and young adults with ME/CFS and their parents express and feel? - Qualitative study in Germany
Source: BMC Public Health. 2026 Sep 29;26:2942. doi: 10.1186/s12889-026-29549-6 (PMC13656452; doi:10.1186/s12889-026-29549-6)
Supplement: Supplementary file 2 [file 12889_2026_29549_MOESM2_ESM.pdf]

Category	Subcategory	Number of participants, n (%)
Highest school-leaving education	Upper secondary school qualification (university entrance qualification)	76%
	Intermediate secondary school qualification	N/A
	Basic secondary school qualification	N/A
	Not reported	N/A
Highest professional qualification	University degree	47%
	University of Applied Sciences degree / Engineering school qualification	N/A
	No professional qualification / not in vocational training	N/A
	Vocational training qualification (apprenticeship)	N/A
	Advanced vocational qualification (e.g., master craftsperson, technician, vocational academy)	N/A
	Not reported	N/A
Employment status	Employed part-time	65%
	Employed full-time	N/A
	Not reported	N/A
Occupational status	Civil servant	29%
	Employee	65%
	Never employed	N/A
	Not reported	N/A
Leadership position	Leadership or supervisory position	41%
	No leadership position	N/A
	Not reported	N/A
Personal net income	> 2833 Euro monthly	88%
	< 2833 Euro monthly	N/A
	Not reported	N/A
Number of persons living in the household	2-3 persons	29%
	≥ 4 persons	71%
Number of persons living in the household ≤ 14 years of age	none	65%
	1-2 persons	35%

Supplementary material *Sociodemographic data of the parental sample*
All frequencies below 5 were reported as N/A to reduce the risk of participant identification and to maintain confidentiality in the context of small cell sizes.
